# Recent applications of immunomodulatory biomaterials for disease immunotherapy

**DOI:** 10.1002/EXP.20210157

**Published:** 2022-05-23

**Authors:** Huaxing Dai, Qin Fan, Chao Wang

**Affiliations:** ^1^ Institute of Functional Nano & Soft Materials (FUNSOM) Jiangsu Key Laboratory for Carbon‐Based Functional Materials & Devices Soochow University 199 Ren'ai Road Suzhou Jiangsu China; ^2^ Key Laboratory for Organic Electronics & Information Displays (KLOEID) Jiangsu Key Laboratory for Biosensors Institute of Advanced Materials (IAM) and School of Materials Science and Engineering Nanjing University of Posts & Telecommunications Nanjing China

**Keywords:** biomaterials, disease treatment, immunomodulatory effect, immunotherapy

## Abstract

Immunotherapy is used to regulate systemic hyperactivation or hypoactivation to treat various diseases. Biomaterial‐based immunotherapy systems can improve therapeutic effects through targeted drug delivery, immunoengineering, etc. However, the immunomodulatory effects of biomaterials themselves cannot be neglected. In this review, we outline biomaterials with immunomodulatory functions discovered in recent years and their applications in disease treatment. These biomaterials can treat inflammation, tumors, or autoimmune diseases by regulating immune cell function, exerting enzyme‐like activity, neutralizing cytokines, etc. The prospects and challenges of biomaterial‐based modulation of immunotherapy are also discussed.

## Graphical abstract

Understanding the immunomodulatory functions of biomaterials themselves is critical for developing safe and effective immunotherapeutic platforms. This review summarizes some representative biomaterials with immunomodulatory functions discovered in recent years and their applications in therapy for inflammation, tumor, and autoimmune diseases, and discusses the challenges for the future development of biomaterials.

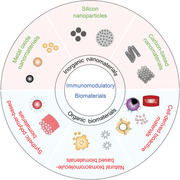

## 1 INTRODUCTION

The immune system is a complex and constantly changing physiological system that works together through a network of connections built by hundreds of cells and molecules.^[^ ^1^, ^2^ ^]^ The immune system, as a defense system, is essential for survival and inextricably associated with pathology and almost every known disease, such as inflammation, cancer, infection, autoimmune diseases, obesity, and aging.^[^ ^3^, ^4^, ^5^, ^6^ ^]^ The occurrence of various diseases is usually accompanied by hypoactivation or hyperactivation of the immune system. For example, cancer cells can evade immune system surveillance by changing their biological characteristics and can suppress the normal function of immune cells in tumors.^[^ ^7^, ^8^, ^9^ ^]^ Aging can also degrade the body's immune system, increasing the susceptibility to infection, cancer, and inflammatory diseases.^[^ ^4^, ^10^ ^]^ Moreover, infectious diseases caused by some viruses (such as AIDS) can attack the human immune system and cause immunodeficiency syndrome.^[^ ^11^, ^12^ ^]^ In addition to the abovementioned immune deficiency‐related diseases, some diseases are caused by immune system overactivation, the most typical of which are autoimmune diseases and allergic reactions.^[^ ^13^, ^14^, ^15^, ^16^ ^]^ In addition, some acute inflammation caused by microbes, such as SARS‐CoV‐2‐induced COVID‐19, is associated with cytokine storms in the lung tissue, leading to respiratory failure and patient death. Therefore, regulating the homeostasis of the immune system is one of the most effective treatments in the biomedical field.

**FIGURE 1:**
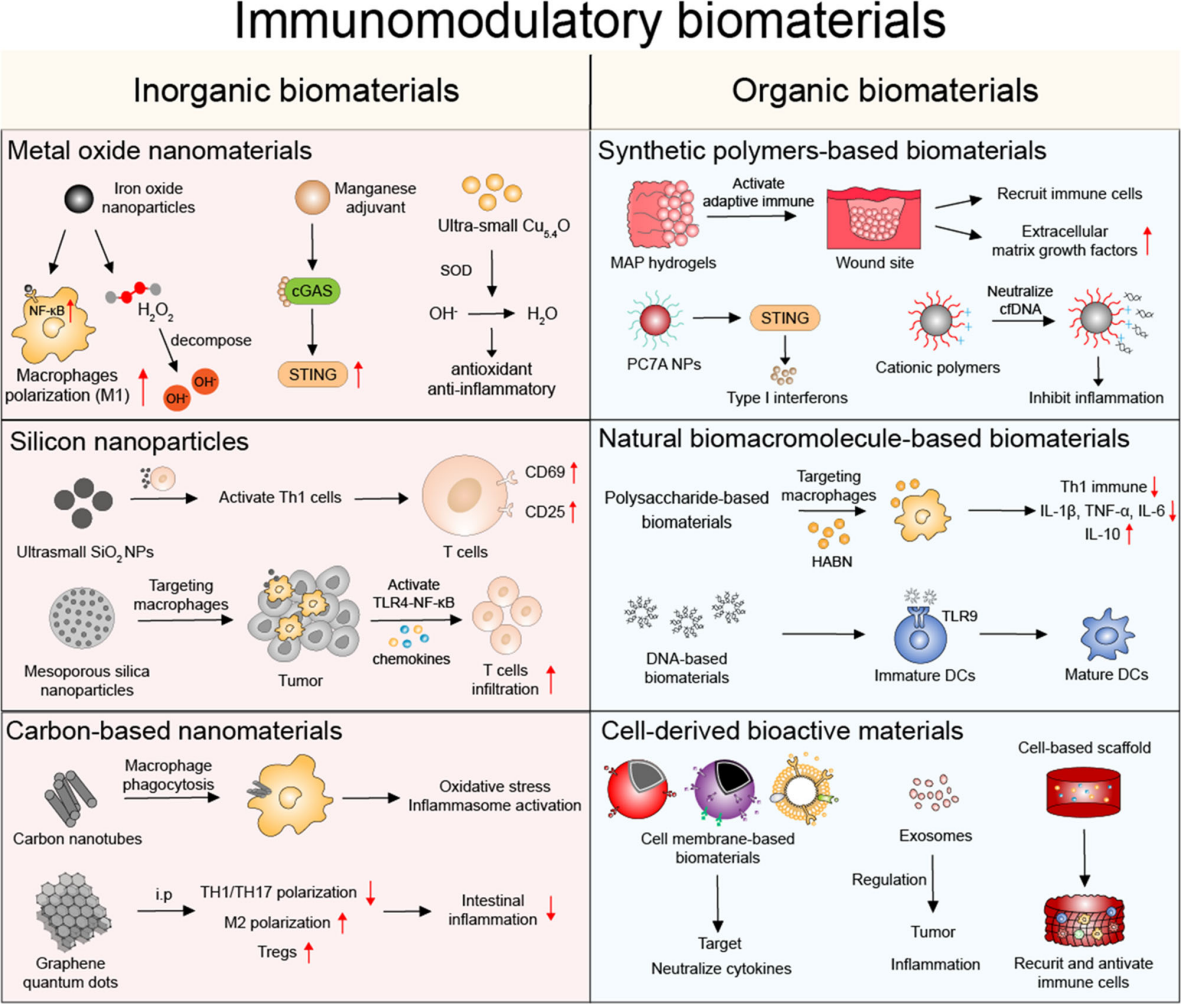
Diagram of representative inorganic and organic biomaterials with immunomodulatory functions

Over the past few decades, many biomaterials have been tailored to construct therapeutic platforms for regulating the immune system against diseases.^[^ ^17^, ^18^ ^]^ Typical biomaterials that interact with biological organisms include inorganic and organic materials. Inorganic biomaterials refer to materials with inorganic substances as the main body.^[^ ^19^ ^]^ Organic materials commonly used in the biomedical field refer to biomaterials based on carbohydrates, nucleic acids, lipids, proteins, synthetic polymers, etc.^[^ ^20^, ^21^, ^22^ ^]^ It is worth noting that among them, a series of biomaterials have been reported to be equipped with immunomodulatory effects, either immunostimulatory or immunosuppressive properties, which have been engineered to develop new immunotherapy strategies for disease treatments. In this review, we focus on the latest research on immunomodulatory biomaterials (Figure 1), including inorganic and organic biomaterials, and provide a perspective on their future opportunities in modulating disease treatment strategies. Understanding the impact of biomaterials on the immune system is a vital prerequisite for developing a safe and effective treatment platform. We believe this review has great significance for the future development of biomaterial‐based treatment platforms.

## 2 INORGANIC NANOMATERIALS

Inorganic nanomaterials are currently one of the most popular nanomaterials in the biomedical field. They generally have stabler physicochemical properties and better controllability than organic materials. To date, inorganic nanomaterials have been widely reported in treatment of cancer, infection, acute kidney injury (AKI), orthopedic and neurological diseases.^[^ ^23^, ^24^, ^25^, ^26^, ^27^, ^28^ ^]^ Notably, as nonself materials, they are definitely recognized as foreign by immune system, resulting in the activation of phagocytes, such as macrophages and DCs.^[^ ^29^ ^]^ This induced inflammation is generally an adverse reaction in healthy bodies^[^ ^30^, ^31^ ^]^ but may beneficial to improve the immunotherapy against tumors. Some inorganic nanomaterials have been used as immunoadjuvants to participate in the construction of an immunotherapy platform. For example, studies have shown that gold nanorods, fullerene derivatives, or graphene oxide derivatives can act as vaccine nanoadjuvants by promoting local inflammation to fight viral infections and tumors.^[^ ^32^, ^33^, ^34^ ^]^ In addition, researchers have developed a series of inorganic nanomaterials with enzymatic catalytic activity and called them “nanozymes.” The size, structure, distribution of nanomaterials, cell internalization method, and environmental pH will all affect their enzyme‐like activity. For example, the chronic inflammatory microenvironment is characterized by elevated levels of reactive oxygen species (ROS), while nanozymes with various activities can regulate the immune response by increasing or reducing ROS around the microenvironment.^[^ ^35^, ^36^ ^]^

### 2.1 Metal oxide nanomaterials

#### 2.1.1 Iron oxide nanoparticles

**FIGURE 2:**
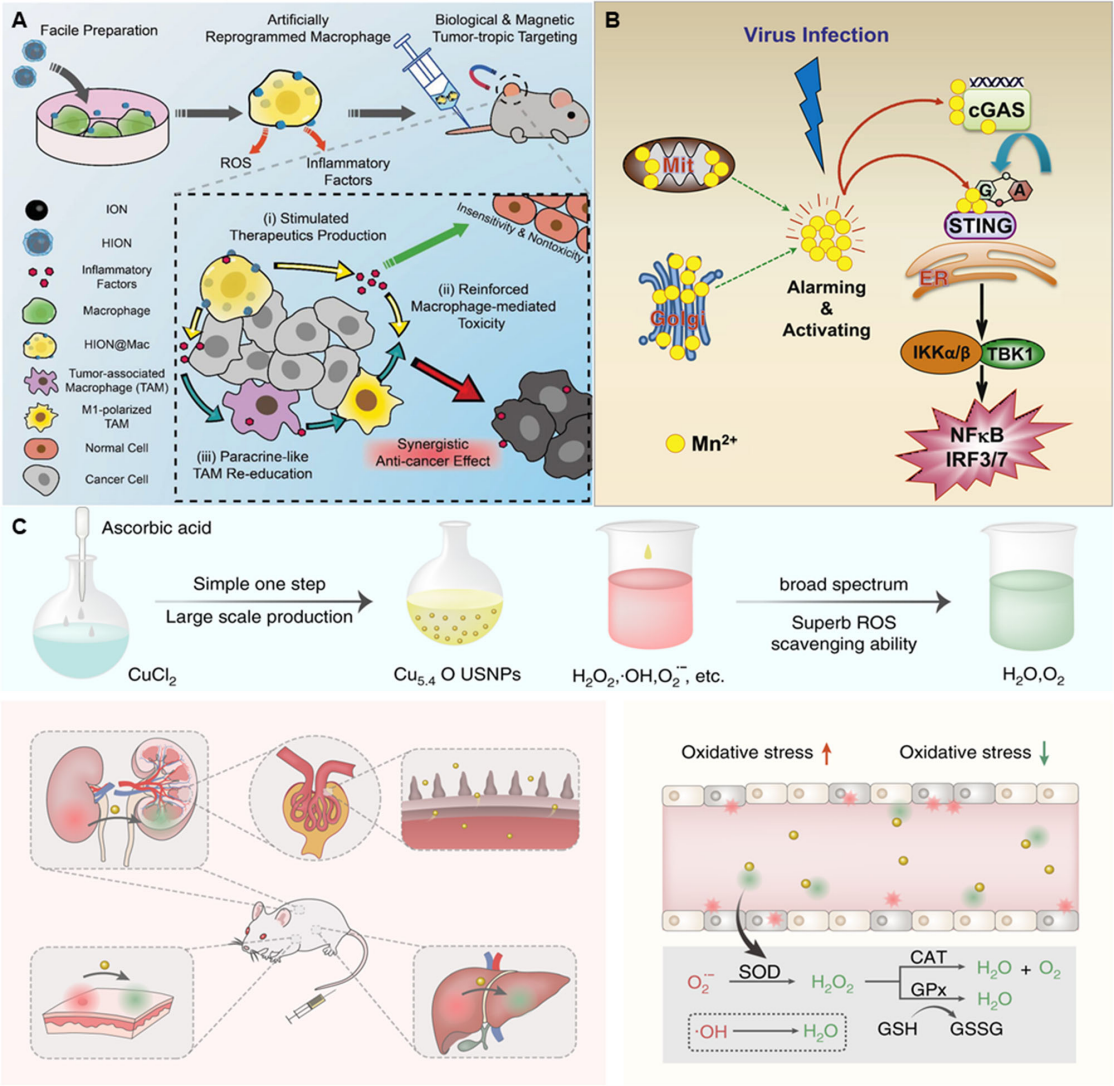
(A) Schematic illustration depicting that artificially reprogrammed HION@Macs target tumors through active chemotaxis and magnet guidance, produce inflammatory factors (such as TNF‐α, NO, and ROS) to suppress tumors, and re‐educate in situ M2 macrophages into a proinflammatory M1 phenotype for synergistic cancer‐specific therapy. Reproduced with permission.^[^ ^43^ ^]^ Copyright 2019, Wiley‐VCH. (B) Mn^2+^ ions enhance the sensitivity of the cGAS‐STING signaling pathway to DNA viruses. Reproduced with permission.^[^ ^55^ ^]^ Copyright 2020, Elsevier. (C) Schematic diagram of the synthesis and mechanism of Cu_5.4_O ultrasmall nanoparticles. Reproduced with permission.^[^ ^69^ ^]^ Copyright 2018, Springer Nature

The Food and Drugs Administration has approved a series of iron oxide nanoparticles for disease diagnosis and treatment, including magnetic resonance contrast agents, treatment of iron deficiency, and construction of a nanovaccine platform.^[^ ^37^, ^38^, ^39^ ^]^ Interestingly, iron oxide nanoparticles have specific immunomodulatory capabilities as reported. Many studies have shown that iron oxide nanoparticles could induce M1 polarization of macrophages, promote the production of ROS, TNF‐α, and NO, and significantly inhibit tumor growth.^[^ ^40^, ^41^, ^42^ ^]^ For example, Li et al. found that after macrophages uptake of iron oxide nanoparticles, increased intracellular iron content can activate the NF‐κB signaling pathway and promote macrophages to initiate TNF‐α‐related immune activation and inflammation (Figure 2A).^[^ ^43^ ^]^ This study shows that iron oxide nanoparticles could induce reprogramming of macrophages to a highly activated state, producing a large number of inflammatory cytokines.

In addition, iron oxide nanoparticles can also regulate the decomposition of hydrogen peroxide through their own enzyme‐like activity, which has been used in the treatment of tumors, inflammation, malaria, dental caries, dental plaque, and other diseases.^[^ ^44^, ^45^, ^46^, ^47^, ^48^, ^49^ ^]^ For example, it has been shown that fenozyme with catalase‐like activity can significantly reduce ROS content in vivo and promote M1‐type polarization of macrophages to kill malaria in the blood, greatly reducing the lethality of experimental cerebral malaria.^[^ ^47^ ^]^ Interestingly, ultrasmall Fe_3_O_4_ nanoparticles exert peroxidase activity under acidic conditions, which can decompose H_2_O_2_ into highly toxic hydroxyl radicals and induce cancer cell death.^[^ ^50^ ^]^ Therefore, iron oxide nanoparticles can be used for the treatment of various diseases by displaying different enzymatic activities.

#### 2.1.2 Manganese‐based nanoadjuvants

As an essential inorganic trace element, manganese participates in synthesizing a variety of enzymes and complex physiological activities. Previous studies have found that manganese dioxide (MnO_2_) has oxidase activity, which can catalyze the decomposition of H_2_O_2_ to enhance cancer immunotherapy.^[^ ^51^, ^52^, ^53^, ^54^ ^]^ Notably, Mn^2+^ ions themselves also have a powerful immunomodulatory effect. Mn^2+^ ions dramatically increased the sensitivity of the cGAS‐STING pathway to recognize microbial and host‐derived DNAs (Figure 2B).^[^ ^55^ ^]^ Lv et al. discovered that Mn^2+^ ions could increase the antigen presentation of dendritic cells and macrophages which activate NK cells and T cells. The combination therapy with aPD1 has achieved good therapeutic effects.^[^ ^56^ ^]^ Based on the above findings, researchers have prepared a manganese adjuvant (manganese salt, MnJ), which can activate cellular immunity, humoral immunity, and mucosal immunity at the same time.^[^ ^57^ ^]^ This study shows that manganese adjuvants have good biological safety and great application potential in the biomedical field. Furthermore, researchers have developed a series of Mn‐based nanomedicines to enhance antibacterial or antitumor immunotherapy.^[^ ^58^, ^59^ ^]^ For example, Sun et al. developed Mn^2+^ ions and STING agonist (cyclic dinucleotide) self‐assembled coordination nanoparticles (CMPs) that are used in cancer treatment.^[^ ^60^ ^]^ The results showed that CMP significantly enhances STING activation and the interferon response, and generates powerful antitumor immunity for refractory tumor model treatment. Moreover, nanovaccines based on manganese adjuvants have also played a good therapeutic effect in the new coronavirus infection model.^[^ ^61^ ^]^ These results show that Mn‐based nanomedicines have strong application potential as adjuvants to improve vaccine or immunotherapy responses.

#### 2.1.3 Other metal‐based nanomaterials

In addition to the typical materials mentioned above, there are also a large number of inorganic materials as reported with immunomodulatory properties. Nanoscale titanium dioxide (TiO_2_) is reported to increase the production of inflammatory cytokines and enhance the maturation of dendritic cells, which activate naïve CD4^+^ T cells.^[^ ^62^ ^]^ TiO_2_ particles with nanospikes can exert mechanical stress on macrophages and dendritic cells, causing K^+^ ion outflow and activation of inflammasomes in the process of phagocytosis, enhancing cellular immunity and the humoral immune response.^[^ ^63^ ^]^ Gold nanoparticles can serve as carriers for constructing vaccines or delivering drugs.^[^ ^64^ ^]^ Study has shown that gold nanoparticles can downregulate the cellular inflammatory response induced by IL‐1β in vivo and in vitro.^[^ ^65^ ^]^ Polymer‐modified gold nanoparticles can significantly enhance tumor immunotherapy without affecting the normal function of B lymphocytes. However, rod‐shaped gold nanoparticles can inhibit the immune response, probably due to their weight that affects the normal function of immune cell membranes.^[^ ^66^ ^]^ Thus, the structure and size of inorganic materials may also affect their immunomodulatory effect.

Some inorganic nanomaterials with catalytic activity are widely used in anti‐inflammatory and antitumor treatments.^[^ ^35^, ^67^, ^68^ ^]^ It is necessary to consider the different physicochemical properties of nanomaterials and the difference in enzyme activity under different conditions (acidic, neutral, alkaline). Liu et al. reported a simple, green, and large‐scale preparation of ultrasmall Cu_5.4_O nanozymes, which can be used as antioxidants to treat various ROS‐related inflammatory diseases (Figure 2C).^[^ ^69^ ^]^ This study shows that ultrasmall Cu_5.4_O has a variety of enzymatic activities, which can effectively eliminate ROS and inhibit the NF‐κB pathway, reduce the levels of the inflammatory factors, and have great therapeutic effects in acute liver injury, diabetes full‐thickness skin defects, and AKI. Metal complexes often have multiple enzyme activities at the same time. For example, copper telluride nanoparticles have glutathione oxidase and peroxidase activities, which can induce oxidative stress in tumors, promote the release of inflammatory factors and trigger innate immune responses.^[^ ^70^ ^]^ The ultrasmall trimetallic (Pd, Cu, Fe) alloy nanozymes also have both peroxidase and glutathione peroxidase activities,^[^ ^71^ ^]^ which can inhibit tumors by intensifying the Fenton reaction. Therefore, by designing inorganic biomaterials with specific enzymatic activities, they can be specific for the treatment of various inflammations and cancers. Similarly, some researchers have found that Prussian blue nanozymes can act as ROS scavengers, exerting antioxidant and anti‐inflammatory effects, and are used to treat acute pancreatitis (AP).^[^ ^72^ ^]^ In fact, there are many studies using the enzyme activity of nanomaterials to develop ROS scavengers for the treatment of inflammatory bowel disease, wound healing, AKI, or acute liver injury.^[^ ^73^, ^74^ ^]^

### 2.2 Silicon nanoparticles

**FIGURE 3:**
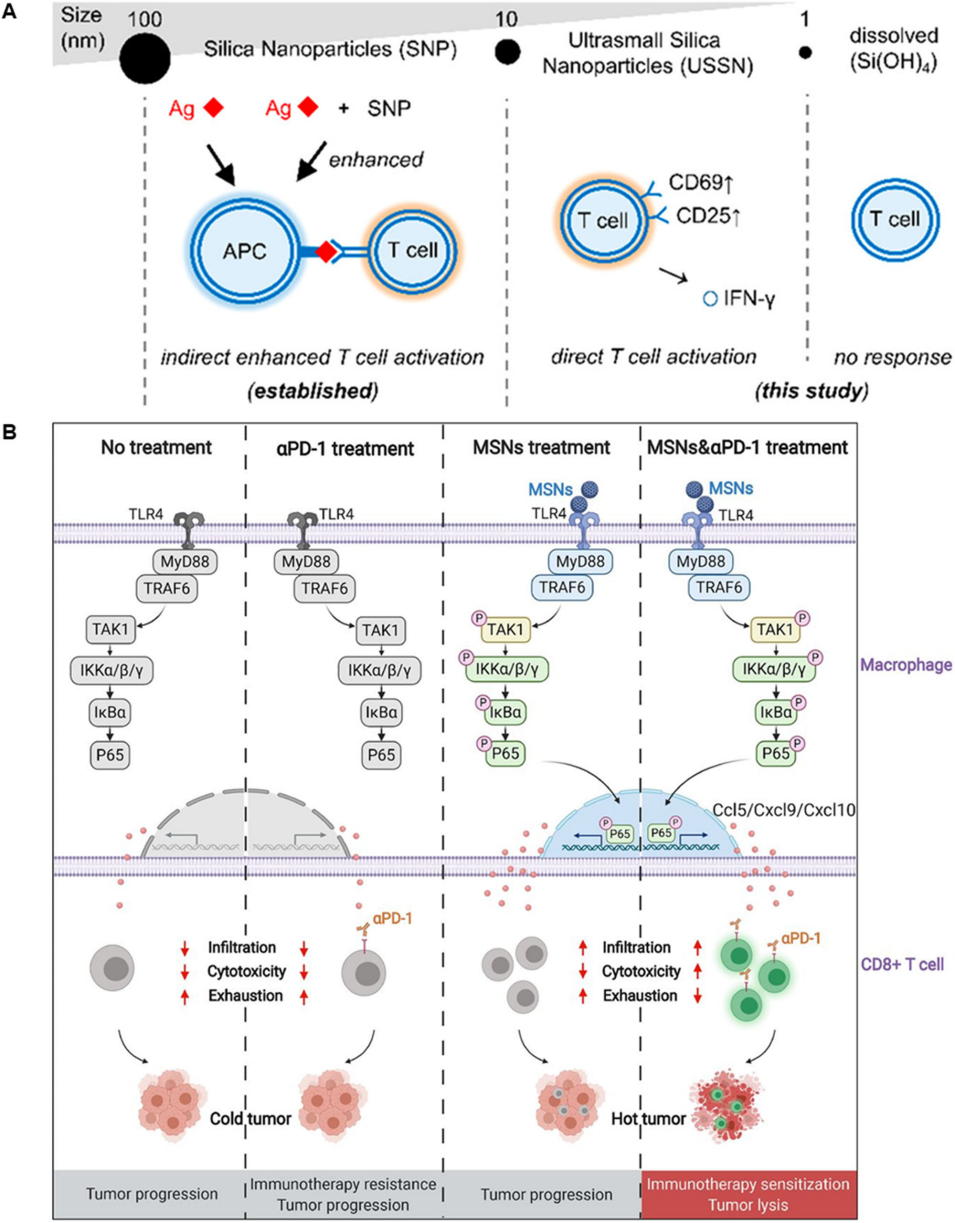
Silicon nanoparticles can regulate T cell activation and improve immune checkpoint therapy. (A) Nonfunctionalized ultrasmall silica nanoparticles directly activate T cells selectively. Reproduced with permission.^[^ ^79^ ^]^ Copyright 2018, American Chemical Society. (B) A single intraperitoneal injection of mesoporous silica nanoparticles (MSNs) can improve the efficacy of PD‐1 in tumor immunotherapy. Reproduced under the terms of the Creative Commons Attribution‐NonCommercial license.^[^ ^90^ ^]^ Copyright 2021, Society for Immunotherapy of Cancer

Silicon nanoparticles have become one of the most promising nanomaterials in recent decades.^[^ ^75^ ^]^ As mentioned before, nonself silica materials, particularly unmodified silica nanoparticles, can activate the immune system to induce inflammation, such as monocytes and the complement system.^[^ ^76^, ^77^, ^78^ ^]^ The inflammation caused by silica nanoparticles in the healthy body may lead to the perturbed immune responses in normal tissues. For example, the enrichment of SiO_2_ NPs in the lungs may lead to the formation of a premetastatic niche in healthy mice, which is manifested as an increase in myeloid cells and T cell dysfunction.^[^ ^31^ ^]^ On the contrary, in the body with the tumor, it is beneficial for immunomodulatory silica nanoparticles to induce the inflammation. Some ultrasmall silica nanoparticles are reported to be effective inducers of immune cell activation (Figure 3A).^[^ ^79^ ^]^ These ultrasmall silica nanoparticles are reported to initiate the activation of T cells through direct particle‐cell interactions related to the dosage of silicon. To date, silica nanoparticles have been widely used to build a multifunctional immunotherapy platform, gradually changing from a simple drug carrier to even a biological regulator, and ultrasmall SiO_2_ NPs have been approved for human clinical trials.^[^ ^80^, ^81^, ^82^, ^83^, ^84^ ^]^

The inflammation induced by silica nanoparticles in tumor‐bearing mice can be utilized against tumors. Hollow mesoporous silica nanoparticles (MSNs) are used as immunoadjuvants to trigger specific immunity against cancer, which significantly enhances the immunotherapeutic effects.^[^ ^85^, ^86^ ^]^ In addition, MSNs are reported to enhance Th1 and Th2 immunity, increase the number of CD4^+^ and CD8^+^ effector memory T cells in the bone marrow, and effectively improve the effect of antitumor immunotherapy.^[^ ^87^, ^88^, ^89^ ^]^ Notably, Sun et al. found that intraperitoneal injection of MSNs could significantly improve the therapeutic effect of PD‐1 antibody (Figure 3B).^[^ ^90^ ^]^ They found that MSNs can target macrophages within tumors and activate the TLR4‐NF‐κB pathway, then increase T cell‐related chemokines secretion (Ccl5/Cxcl9/Cxcl10) to recruit T cells in tumors, effectively overcoming PD‐1 antibody resistance. Because tumors with a “cold” immunological phenotype show a large number of immunosuppressive cells (e.g., Tregs and TAMs) and a limited number of tumor‐infiltrating lymphocytes (TILs), using the immunomodulatory effect of silicon nanoparticles to improving the local inflammation may be a potential solution.

### 2.3 Carbon‐based nanomaterials

Using carbon nanomaterials (C‐BNMs) (e.g., carbon nanotubes, quantum dots, or graphene oxide) as therapeutic platforms has been reported.^[^ ^91^, ^92^, ^93^, ^94^, ^95^ ^]^ As a kind of refractory nanomaterial, C‐BNMs could enhance the phagocytosis of monocytes and accelerate the differentiation of monocytes into macrophages.^[^ ^96^ ^]^ But people pay more attention to their carcinogenicity, such as the carbon nanotubes. Lu et al. reported that long‐term exposure to multiwalled carbon nanotubes could enhance the invasiveness of breast cancer cells implanted in situ and accelerate the lung colonization of cancer.^[^ ^97^ ^]^ Therefore, although carbon nanotubes have unique physicochemical properties and have capacity to induce strong inflammation, their carcinogenicity greatly limits their application in many fields.

**FIGURE 4:**
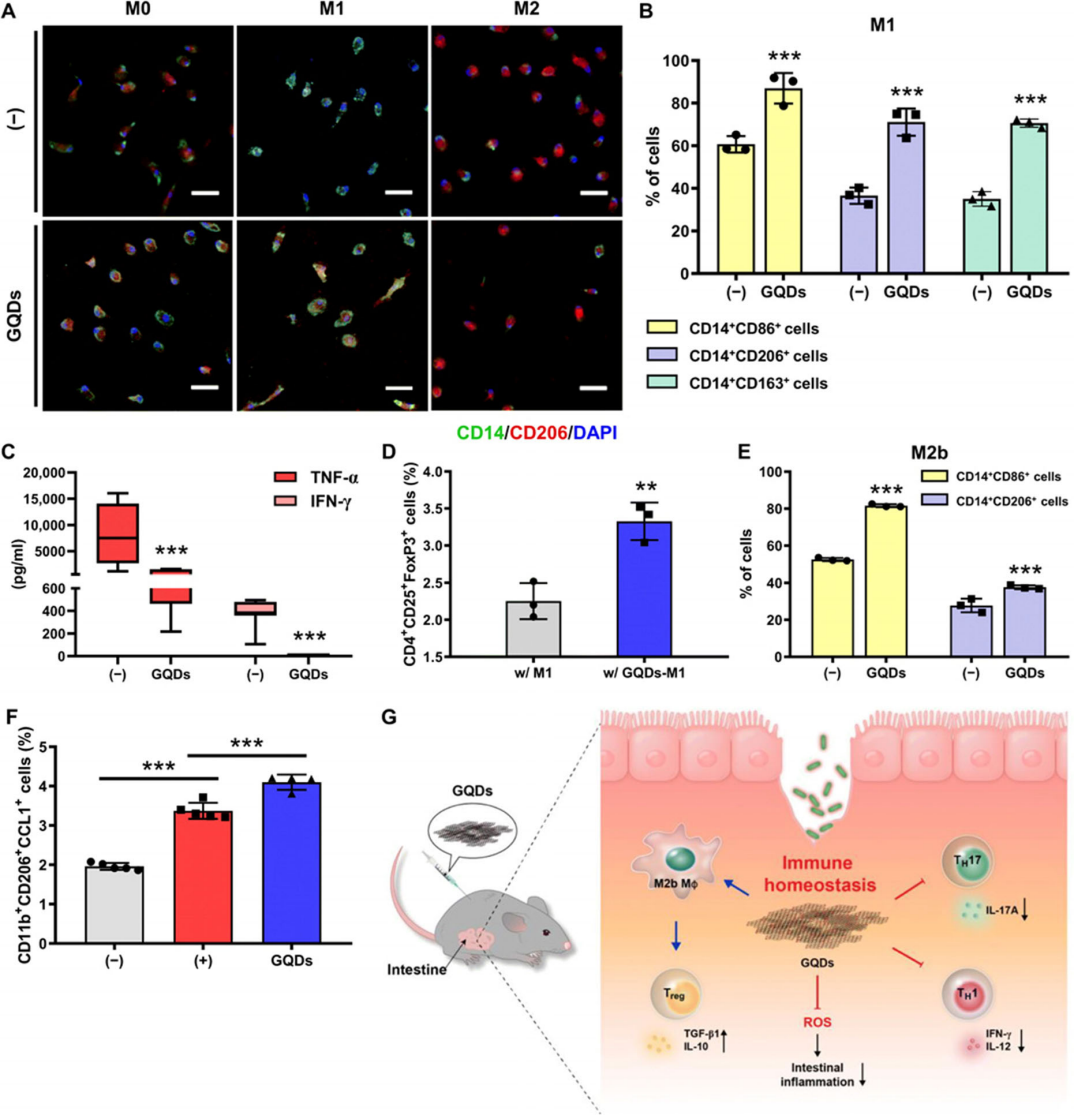
Graphene quantum dots (GQDs) promote M2 polarization of macrophages. (A,B) Primary CD14^+^ macrophage‐like cells are polarized into M0‐, M1‐, and M2‐type cells in the presence of GQDs. (C) Cytokine concentration in the supernatant of M1 macrophages. (D) M1‐induced cells in the presence of GQDs were cocultured with naïve CD4^+^ T cells supplemented with IL‐2 and TGF‐β1, and the proportions of Tregs were investigated by flow cytometry. (E) Flow cytometry analysis of M2b polarization in vivo. (F) Flow cytometry to evaluate the proportion of M2b macrophages in the peritoneum of mice with chronic colitis. (G) Schematic diagram of the mechanism of action of GQDs. Reproduced under the terms of the Creative Commons Attribution‐NonCommercial license.^[^ ^102^ ^]^ Copyright 2020, American Association for the Advancement of Science

In addition to carbon nanotubes, graphene oxide has also been reported to interact with macrophages and T cells in vitro and can act as a natural antioxidant, reducing M1 polarization by reducing ROS in macrophages.^[^ ^98^, ^99^ ^]^ Graphene oxide can also attenuate the TH2 immune response in a mouse asthma model.^[^ ^100^ ^]^ Its derivative graphene quantum dots (GQDs) have good biocompatibility and antioxidant properties that can be used in inflammatory disease (such as hepatitis) treatments.^[^ ^101^ ^]^ Lee et al. evaluated the effects of GQDs on intestinal diseases therapy. The results showed that in acute and chronic enteritis mouse models, intraperitoneal injection of GQDs inhibited TH1/TH17 polarization, promoted macrophage M2 polarization, increased the Treg ratio, and effectively alleviated the inflammatory response in the intestine (Figure 4).^[^ ^102^ ^]^ In addition, researchers have found that graphene quantum dots can penetrate the blood–brain barrier and inhibit the formation of fibrotic aggregates of α‐synuclein in Parkinson's disease.^[^ ^103^ ^]^ In addition to GQDs, some studies have shown that water‐soluble quantum dots can activate the NF‐κB signaling pathway and exert antitumor, antiviral, and anti‐inflammatory effects by regulating immunity.^[^ ^104^, ^105^ ^]^

Therefore, inorganic nanomaterials with immunomodulatory effects have certain potential for development in the biomedical field, but there are still many problems that urgently need to be solved. For example, the role of inorganic nanomaterials can be adjuvant (antitumor) or cancerogen (promote tumor), both of which are caused by inflammation. The underline mechanism is still not very clear. Meanwhile, most reported inorganic nanomaterials to attenuate inflammatory response was by enzyme‐like activity. How to regulate the complex enzyme activity of inorganic nanomaterials requires further research.

## 3 ORGANIC BIOMATERIALS

Many organic biomaterials show immunomodulatory properties when they come into contact with biological organisms.^[^ ^106^, ^107^ ^]^ Organic biomaterials with immunomodulatory functions can be divided into synthetic polymer materials, natural biomacromolecule materials, and cell‐derived bioactive materials in this paper.^[^ ^107^, ^108^ ^]^

### 3.1 Synthetic polymer‐based biomaterials

Synthetic polymers are ubiquitous in medicine. Generally, synthetic polymers are used as inert stents or carriers to deliver drugs.^[^ ^109^, ^110^ ^]^ These polymers have been proven to dramatically improve the therapeutic effect by reducing off‐target effect or achieving sustained drug release. With the advancement of immunotherapy technology, polymers with specific biological functions can be prepared by conjugating some adjuvants with inert polymers or directly chemical structure modifications.^[^ ^111^, ^112^, ^113^ ^]^

**FIGURE 5:**
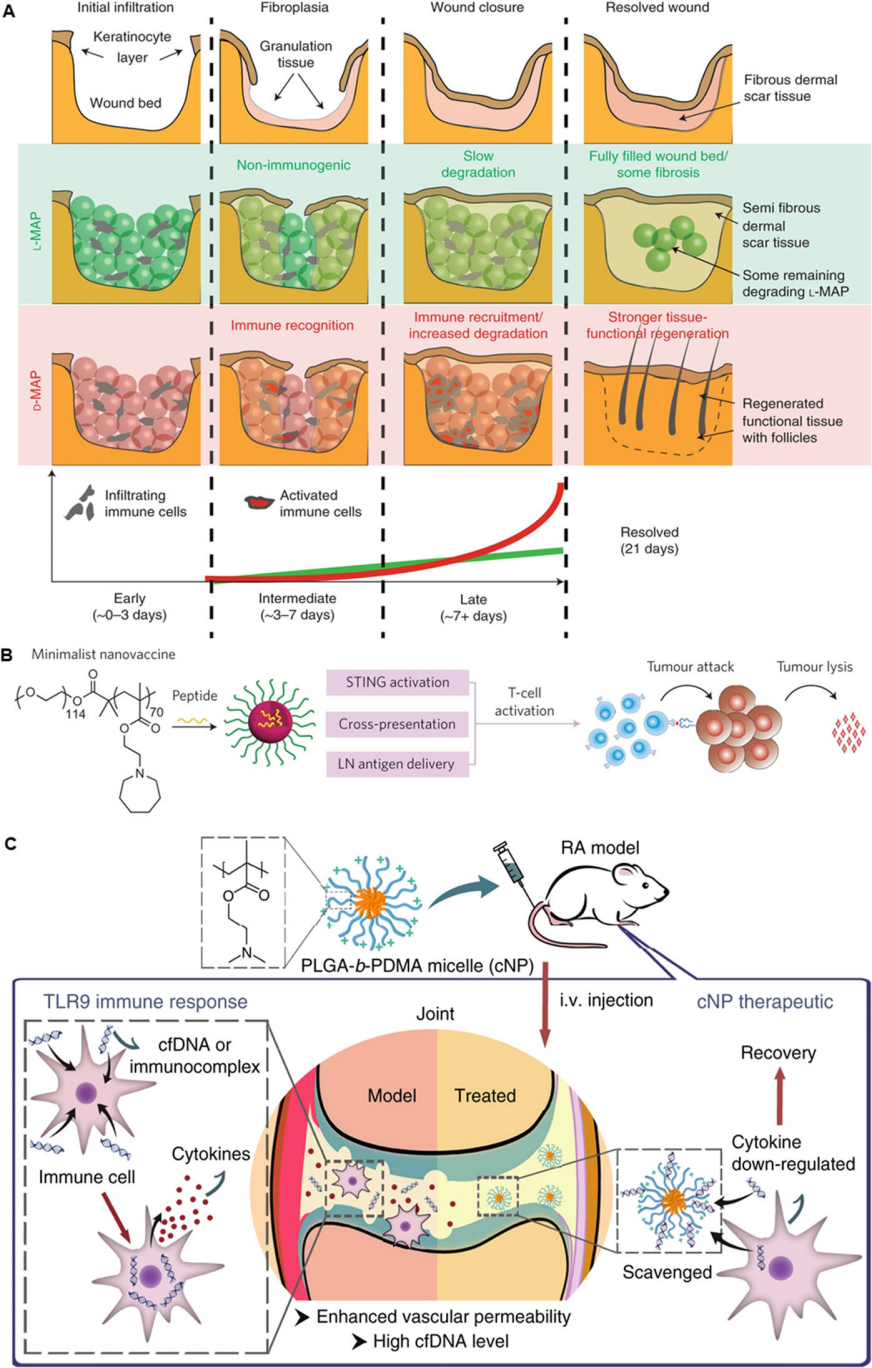
(A) The use of L‐ or D‐MAP in wound healing models. In the case of d‐MAP, the hydrogel activates the adaptive immune system, leading to tissue remodeling and skin regeneration as the adaptive immune system degrades the D‐MAP scaffold. Reproduced with permission.^[^ ^117^ ^]^ Copyright 2021, Springer Nature. (B) Schematic of the minimalist design of the PC7A nanovaccine. Reproduced with permission.^[^ ^121^ ^]^ Copyright 2017, Springer Nature. (C) Cationic nanoparticles have high free DNA binding capacity, which can effectively inhibit the activation of TLR9 and inhibit the inflammatory response of rheumatoid arthritis. Reproduced with permission.^[^ ^125^ ^]^ Copyright 2018, Springer Nature

During the past few decades, polymers have been modified as functional polymers in anti‐inflammatory, anti‐infection, and tissue regeneration.^[^ ^114^, ^115^, ^116^ ^]^ For example, Scumpia et al. found that a degradable gel scaffold composed of microporous annealed particles (MAPs) could activate the type‐2 adaptive immune response (Figure 5A).^[^ ^117^ ^]^ In this study, the researchers flipped the amino acid structure to obtain a D‐peptide cross‐linked MAP hydrogel, which could rapidly degrade at the wound site and activate the adaptive immune system. Immune cells recruited to the wound release large amounts of extracellular matrix growth factors to induce skin regeneration and healing. Meanwhile, hydrogels are one of the most popular polymers for cancer immunotherapy.^[^ ^118^, ^119^, ^120^ ^]^ Gao et al. developed methacrylate‐based acid‐responsive nanoparticles (PC7A) that could target lymph nodes for antigen presentation and activate the STING pathway to promote the release of type I interferons (Figure 5B).^[^ ^121^ ^]^ Researchers also optimized the vaccine system and synthesized a degradable acid‐responsive polymer adjuvant (PSC7A) instead of polycarbonate to improve the system safety.^[^ ^122^ ^]^

By designing the structure of nanoparticles and selectively changing the shape, size, charge, and surface functionalization of the nanoparticles, specific immunomodulatory effects can be achieved.^[^ ^123^, ^124^ ^]^ For example, Chen et al. reported that cationic nanoparticles (cNPs) synthesized by the diblock copolymer of PLGA and PDMA can clear cell‐free DNA (cfDNA) and then downregulate the activation of primary synovial fluid monocytes and fibroblast‐like synovial cells (Figure 5C).^[^ ^125^ ^]^ The sites of rheumatoid arthritis (RA) show enhanced vascular permeability and high cellular free DNA levels. Studies also showed that cfDNA can be absorbed by cationic polymers, relieve the inflammatory response caused by acute liver injury, and relieve systemic lupus erythematosus (SLE).^[^ ^126^, ^127^ ^]^ Another study reported that some PEI‐M polymers obtained by mixing azole small molecules with polyethyleneimine (PEI) could stimulate DC 2.4 immune cells to secrete IFN‐β.^[^ ^128^ ^]^ In addition, Xu et al. found that both designed fluoropolymers (F7‐PEI and F13‐PEI) could stimulate the expression of costimulatory molecules such as CD80 after co‐incubation with mouse bone marrow‐derived dendritic cells.^[^ ^129^ ^]^ Therefore, the design of the structure of polymers is one of the potential strategies for regulating immune response.

### 3.2 Natural biomacromolecule‐based biomaterials

As natural components of organisms, biomacromolecules (e.g., polysaccharides, nucleic acids) usually exhibit high biological activity and have certain immunomodulatory functions.^[^ ^130^ ^]^ Biomaterials based on these natural biomacromolecules inherit their immunomodulatory functions and have been widely used in the development of immunotherapeutics for various diseases.^[^ ^131^ ^]^

**FIGURE 6:**
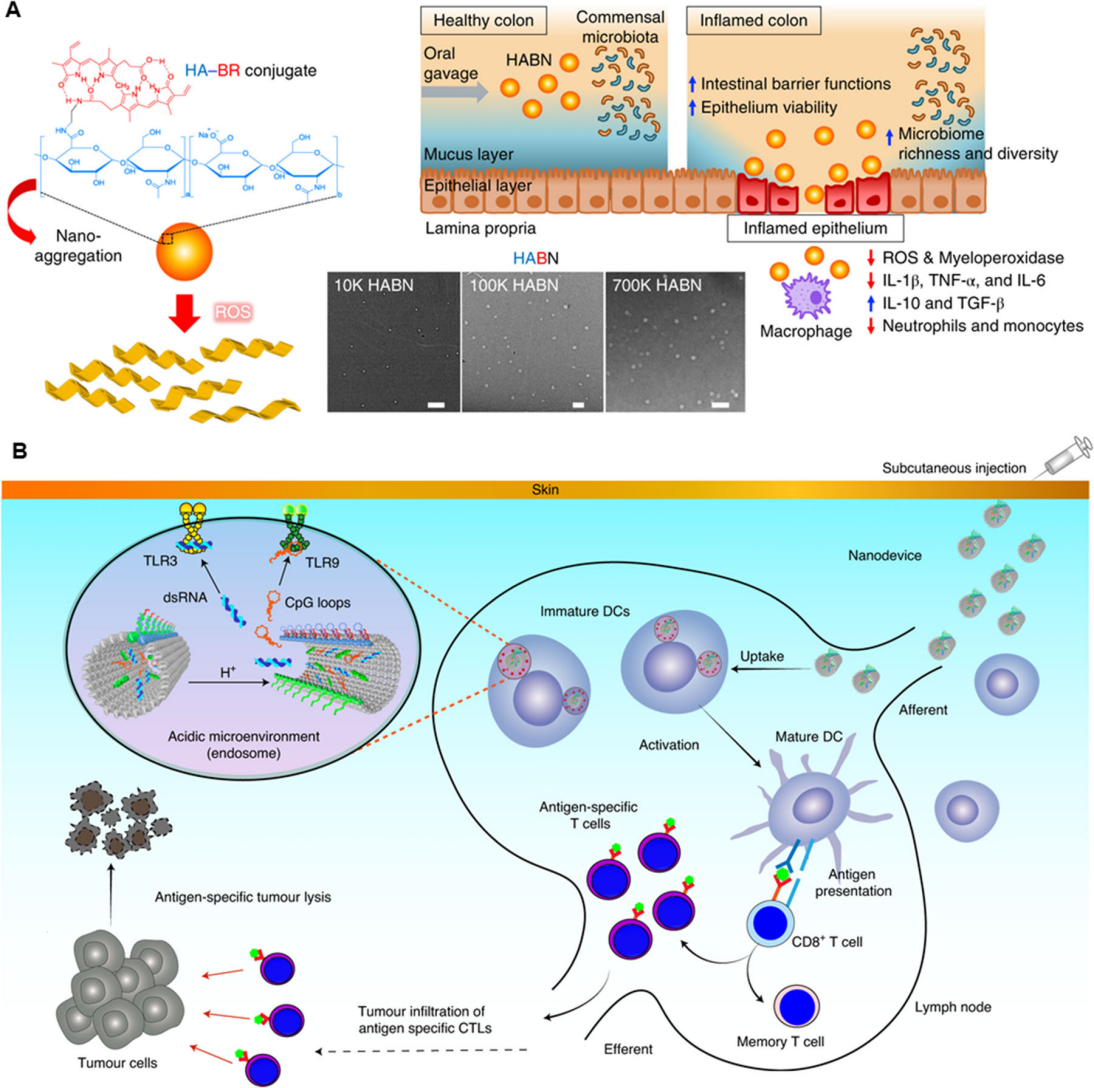
(A) Schematic diagram and TEM image of HABN obtained by self‐assembly of hyaluronic acid (HA)‐bilirubin (BR). HABN accumulates in the inflamed colon and has a therapeutic effect on acute colitis. Reproduced with permission.^[^ ^140^ ^]^ Copyright 2020, Springer Nature. (B) Schematic representation of the utilization of DNA nanodevices for efficient cancer immunotherapy. Reproduced with permission.^[^ ^146^ ^]^ Copyright 2020, Springer Nature

Polysaccharides are naturally occurring biomacromolecular polymers with immunomodulatory properties.^[^ ^132^, ^133^ ^]^ Ganoderma lucidum polysaccharide, lentinan, poria polysaccharide, etc., especially fungal polysaccharides, have the ability of enhancing immunity and antitumor activity.^[^ ^134^, ^135^, ^136^, ^137^ ^]^ Han et al. prepared a polysaccharide‐based inulin gel that can effectively regulate the intestinal flora, promote the proliferation of symbiotic microorganisms, and induce the production of memory CD8^+^ T cells.^[^ ^138^ ^]^ Yeast cell wall includes polysaccharides, such as β‐glucan which has been considered as ‘danger signals’ to activate potent, multiepitope immune response in our body. Xu et al. fabricate nano‐formulations derived from yeast cell wall (YCW NPs) that can remodel the immunosuppressive microenvironment in tumor and tumor‐draining lymph nodes to suppress tumor growth.^[^ ^133^ ^]^ Hyaluronic acid (HA) has typical immunomodulatory effects as an acidic mucopolysaccharide. It is reported that low molecular weight HA stimulates the activation of M1‐like macrophages and increased release of nitric oxide while high molecular weight HA stimulates the alternate activation of M2‐like macrophages, upregulating the expression of Arg1 and IL10.^[^ ^139^ ^]^ In addition, studies have reported that HA could regulate proinflammatory macrophages, inhibit the Th1 immune response, and then change the structure of the intestinal flora to treat inflammatory bowel disease (Figure 6A).^[^ ^140^, ^141^ ^]^

Nucleic acids are another naturally sourced biomaterials for biomedical engineering beyond traditional genetic information storage. Owing to its high programmability and excellent homology, it is feasible to construct various sophisticated structures for drug delivery via simple base‐pairing rules.^[^ ^142^, ^143^ ^]^ Well‐designed DNA carriers showed unique advantages in tumor targeting and delivering nucleic acid immunomodulators, reducing immune‐related toxic side effects. For example, cytosine‐phosphate‐guanine (CpG) oligonucleotides, convenient Toll‐like receptor 9 (TLR9) agonists for activating host immunotherapy, have been loaded onto DNA carriers involving DNA tetrahedra, DNA origami, DNA nanocoons, etc. Recent studies reported that CpG strands integrated with DNA tetrahedra or DNA origami showed a great ability to internalize into macrophages and achieve higher therapeutic outcomes.^[^ ^144^, ^145^ ^]^ Liu and coworkers developed a DNA nanorobot by precisely decorating rectangular DNA origami with different Toll‐like receptor (TLR) agonist and antigen (peptide) capture chains (Figure 6B).^[^ ^146^ ^]^ This DNA nanorobot can be transported to draining lymph nodes (dLNs) to avoid interference by extracellular ribonucleases, achieving effective tumor suppression and preventing tumor recurrence by producing long‐lasting immune memory of specific tumor antigens. Moreover, DNA origami itself has the potential to activate innate immunity through non‐TLR9‐mediated pathways, which should be considered during the design of drug delivery vehicles.^[^ ^144^ ^]^

## 4 CELL‐DERIVED BIOACTIVE MATERIALS

### 4.1 Cell membrane‐based biomaterials

The cell membrane‐based biomaterials can be derived from many types of cells (e.g., red blood cells, immune cells, and cancer cells) which are usually rich in biologically active proteins and phospholipids.^[^ ^147^ ^]^ Researchers have developed a series of membrane‐encapsulated nanoparticles during the past few decades that can effectively play a therapeutic role.^[^ ^21^, ^148^ ^]^ For example, red blood cell membrane (RBCM) modification can significantly increase the blood circulation time of nanoparticles and enhance tumor targeting ability.^[^ ^149^, ^150^, ^151^ ^]^ In addition, RBC‐NPs are reported to target eliminating pathological antibodies that play an essential role in type II hypersensitivity and alleviate the immune response.^[^ ^152^ ^]^ Genetically engineered cell membrane‐based nanoparticles can also be used as drug carriers or immunomodulators to participate in disease treatment.^[^ ^153^, ^154^ ^]^ For example, Shen et al. obtained MSC‐PD‐L1^+^ nanoparticles by wrapping PD‐L1^+^ mesenchymal stem cell (MSC) membranes on the surface of PLGA nanoparticles.^[^ ^155^ ^]^ MSCs are multifunctional stem cells of the body that can regulate the function of innate and acquired immune systems.^[^ ^156^, ^157^ ^]^ Thus, intravenous MSC‐PD‐L1^+^ NPs can be used as immunosuppressive nanoparticles to inhibit the excessive activation of liver macrophages and T cells and reduce liver irAEs caused by high‐dose immune checkpoint blocking (ICB) antibody therapy.

**FIGURE 7:**
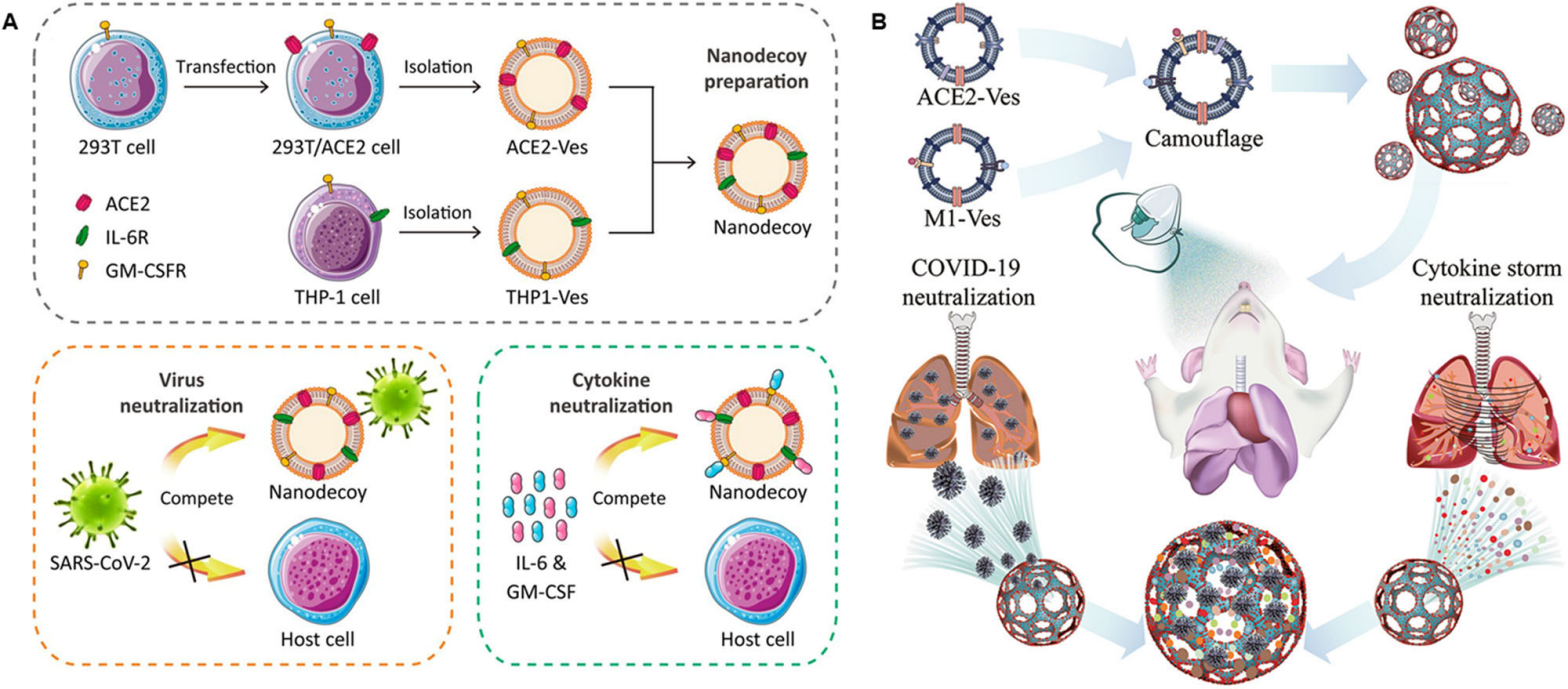
(A) Preparation of anti‐COVID‐19 nanodecoys by fusing cellular membrane nanovesicles derived from genetically edited 293T/ACE2 and THP‐1 cells. Nanodecoys fight COVID‐19 infection by neutralizing SARS‐CoV‐2 and inflammatory cytokines. Reproduced under the terms of the Creative Commons Attribution‐NonCommercial license.^[^ ^164^ ^]^ Copyright 2020, United States National Academy of Sciences. (B) Schematic illustration of the inhaled ACE2‐engineered microfluidic microsphere for neutralization of COVID‐19 and calming of the cytokine storm. Reproduced with permission.^[^ ^167^ ^]^ Copyright 2020, Elsevier

A series of recent studies have shown that macrophage membrane‐coated NPs can participate in the construction of inflammation and cancer immunotherapy platforms.^[^ ^158^ ^]^ For example, Gao et al. found that nanoparticles wrapped by macrophage membranes can absorb proinflammatory cytokines through receptors on the membrane surface and alleviate local inflammation.^[^ ^159^ ^]^ Their results showed that macrophage membrane‐encapsulated nanoparticles could utilize the synergistic effect of membranes and drugs to dramatically prevent the progression of atherosclerosis. In addition, MΦ‐NPs made of macrophage cell membrane‐coated polymers can inhibit endotoxins triggered immune activation and reduce the levels of proinflammatory cytokines.^[^ ^160^ ^]^ This detoxification strategy gave the infected mice a significant survival advantage. Notably, nanosponges prepared using macrophage membrane‐encapsulated polymers are reported to quell coronavirus‐induced cytokine storms by absorbing inflammatory cytokine proteins.^[^ ^161^ ^]^ Furthermore, cell membrane‐based fusion cellular vesicles can also be used to suppress SARS‐CoV‐2 infection or enhance cancer immunotherapy.^[^ ^162^, ^163^ ^]^ To suppress the cytokine storm caused by viral infection, Rao et al. developed a cell membrane‐based nanodecoy composed of the fusion of genetically engineered cell membrane vesicles expressing the angiotensin‐converting enzyme 2 (ACE2) receptor and monocyte membrane vesicles (Figure 7A).^[^ ^164^ ^]^ Because SARS‐CoV‐2 normally enters target cells through ACE2 receptors,^[^ ^165^ ^]^ the nanodecoy can competitively bind to viruses and neutralize cytokines through monocyte membrane receptors, protecting the host against SARS‐CoV‐2 infection. Well‐designed nanocatchers with ACE2 receptor‐overexpressing cell membranes mixed with hyaluronic acid (HA) can significantly prolong its residence time in the lung and exert longer‐lasting virus clearance effects.^[^ ^166^ ^]^ Similarly, the microspheres obtained by fusing the M1 macrophage membrane with ACE2 receptor‐overexpressing cell membranes can also adsorb viruses and neutralize cytokines after inhalation (Figure 7B).^[^ ^167^ ^]^

To enhance cancer immunotherapy, Meng et al. designed a genetically engineered fusion cell membrane vesicle with high expression of both SIRPα and PD‐1, which enhanced antigen presentation and T cell activation by competing with host immune cells for binding to CD47 and PD‐L1 on tumor cells.^[^ ^163^ ^]^ The tumor cell membrane can also be fused with RBCMs, effectively activating various immune cells in vivo and generating a robust antitumor immune response.^[^ ^168^ ^]^ In addition, it has been reported that bacterial outer membrane vesicles can promote the differentiation of macrophages to the M1 type and increase T cell infiltration in tumors,^[^ ^169^ ^]^ which can also be used for antitumor therapy.

**FIGURE 8:**
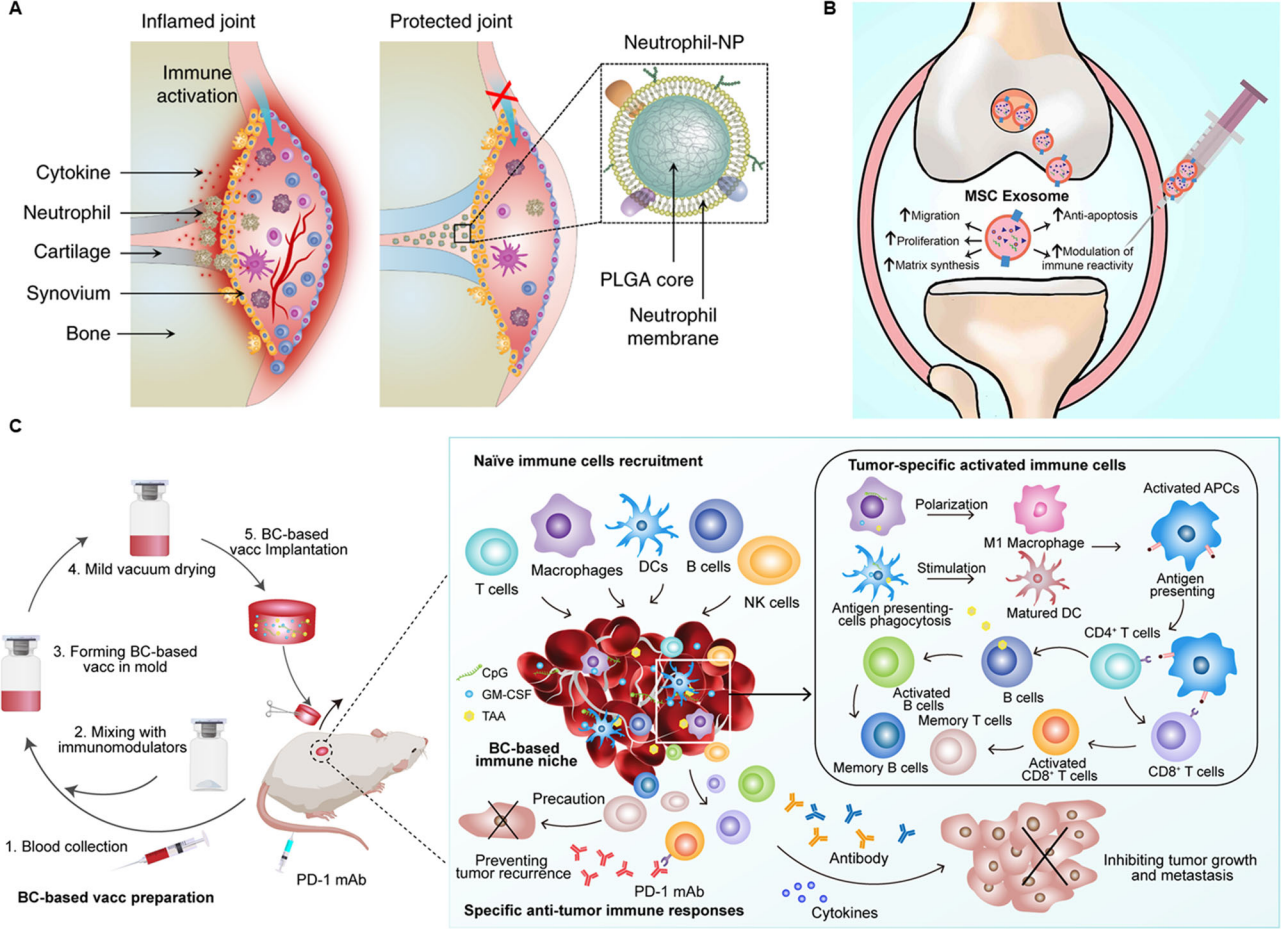
(A) A schematic diagram of neutrophil membrane‐coated nanoparticles inhibiting synovial inflammation and improving joint destruction in inflammatory arthritis. Reproduced with permission.^[^ ^173^ ^]^ Copyright 2018, Springer Nature. (B) Mesenchymal stem cell exosomes promote cartilage repair and regeneration through various mechanisms, such as promoting cartilage proliferation, migration, and matrix synthesis, reducing cell apoptosis, and regulating the immune response. Reproduced with permission.^[^ ^195^ ^]^ Copyright 2018, Elsevier. (C) Schematic diagram of the preparation and mechanism of implantable blood clot vaccine. Reproduced under the terms of the Creative Commons Attribution‐NonCommercial license.^[^ ^215^ ^]^ Copyright 2020, American Association for the Advancement of Science

Excessive activation of the immune system is also accompanied by allergies,^[^ ^170^ ^]^ SLE,^[^ ^171^ ^]^ or rheumatoid arthritis.^[^ ^172^ ^]^ Developing an immunosuppressive treatment platform to neutralize the body's excessive immune response is one effective treatment strategy. Immune cells, especially neutrophils, are critical in inflammation and the oxidative microenvironment. The infiltration of neutrophils is accompanied by an increase in local inflammatory cytokine concentrations. To this end, Zhang et al. developed a neutrophil membrane‐encapsulated nanoparticle with PLGA as a support inside, relying on the neutrophil receptor‐cytokine specific combination to suppress inflammation to treat rheumatoid arthritis (Figure 8A).^[^ ^173^ ^]^ In addition, the neutrophil membrane can destroy the proliferation and tumor metastasis of MDSCs by neutralizing cytokines,^[^ ^174^ ^]^ and it can also be used to improve the oxidative microenvironment after traumatic spinal cord injury (SCI).^[^ ^175^ ^]^

### 4.2 Exosomes

Exosomes are extracellular vesicles rich in specific nucleic acids, proteins, lipids, and carbohydrate complexes.^[^ ^176^ ^]^ In recent years, exosomes have developed rapidly, mainly focusing on exosome‐related drug delivery systems and disease treatments.^[^ ^177^, ^178^ ^]^ In fact, in addition to drug delivery platforms, natural exosomes themselves also have immunomodulatory effects,^[^ ^179^, ^180^ ^]^ which mainly depend on the protein and nucleic acid components. To date, the main studies are exosomes derived from macrophages, dendritic cells, MSCs, granulocytes, tumor cells, and exosomes of plant origin.

Macrophage‐derived exosomes are related to their own phenotypes. Macrophage exosomes from different sources have different biological functions in different diseases (e.g., tumors, inflammation, diabetes, or atherosclerosis).^[^ ^181^ ^]^ It can be used as a carrier for drug delivery and can also exert immunomodulatory functions by itself. Macrophage exosomes can enhance the T cell immune response,^[^ ^182^ ^]^ M1 macrophage exosomes can regulate the M1 polarization of TAMs to inhibit tumor growth,^[^ ^183^ ^]^ and M2 macrophage exosomes (M2 Exos) have anti‐inflammatory effects and can be used for atherosclerosis treatment.^[^ ^184^ ^]^ The exosomes of macrophages can also repolarize M2 tumor‐associated macrophages to the M1 type by releasing proinflammatory cytokines.^[^ ^185^ ^]^ More interestingly, miR‐690 in M2 Exos has an insulin‐sensitizing effect, which can be used to reduce obesity‐induced insulin resistance.^[^ ^186^ ^]^

Dendritic cells are the main antigen‐presenting cells. Like DCs, DC‐derived exosomes may contain MHC peptide complexes, costimulatory molecules, and other components that interact with immune cells.^[^ ^187^ ^]^ Thus, DC‐derived exosomes can be used to participate in the construction of nanovaccines or cancer treatment. It has been shown that extracted engineered dendritic cell exosomes expressing α‐fetoprotein can trigger an antigen‐specific immune response in orthotopic liver cancer mice, which is manifested by increased numbers of CD8^+^ T cells, increased levels of IFN‐γ and IL‐2, and decreased numbers of Treg cells, resulting in a significant inhibition of tumor growth.^[^ ^188^ ^]^ Moreover, DC‐derived extracellular vesicles can also regulate monocyte differentiation and T cell activation.^[^ ^189^, ^190^ ^]^

MSCs are a special type of cell that can support or inhibit the development of tumors by releasing exosomes.^[^ ^191^, ^192^ ^]^ Therefore, many studies have used genetic engineering technology to prepare engineered MSC exosomes for the treatment of specific diseases.^[^ ^193^ ^]^ Xu et al. developed genetically engineered MSC‐derived extracellular vesicles for the treatment of psoriasis and colitis.^[^ ^194^ ^]^ In this study, MSC‐derived extracellular vesicles with high expression of PD‐L1 were constructed using lentiviral transfection technology. PD‐L1 combined with PD‐1 on activated immune cells and inhibited excessive cell activation. Additionally, MSC‐derived exosomes (MSC‐Exos) can increase the proliferation and infiltration of chondrocytes by activating the serine‐threonine kinase (AKT) and extracellular regulatory protein kinase (ERK) signaling pathways, promote the M2 polarization of macrophages and downregulate the expression of inflammatory cytokines (Figure 8B).^[^ ^195^ ^]^ MSC‐Exos can also regulate colon and lung macrophages, effectively reducing inflammation, and MSC‐Exo‐treated macrophages have good resistance to restimulation of inflammation, which can be used to treat inflammatory bowel disease and pulmonary fibrosis.^[^ ^196^, ^197^ ^]^

Granulocyte‐derived exosomes regulate the differentiation of monocytes^[^ ^198^ ^]^ and often promote the occurrence of many lung diseases. For example, neutrophil‐derived pathogenic exosomes can promote the destruction of the extracellular matrix and lead to chronic obstructive pulmonary disease.^[^ ^199^ ^]^ Based on the inflammatory chemotaxis of granulocytes, granulocyte exosomes can be used as carriers to deliver antitumor drugs. Neutrophil‐derived exosomes (NEs‐Exos) can be loaded with doxorubicin. In a glioma model, NEs‐Exos can carry drugs across the blood–brain barrier and significantly inhibit glioma.^[^ ^200^ ^]^ Similarly, tumor cells can also regulate tumor growth and metastasis by releasing exosomes, called “immune escape.” Several studies have shown that in the advanced stage of cancer, tumor cells can release exosomes to establish an immunosuppressive microenvironment before metastasis in the distal end and promote the occurrence of metastasis.^[^ ^201^, ^202^, ^203^ ^]^

Some natural plant exosomes also have immunomodulatory effects in addition to the exosomes mentioned above.^[^ ^204^ ^]^ Exosomes extracted from green tea can target macrophages in the intestines, reduce the production of ROS and inflammatory factors, and be used to treat inflammatory bowel disease.^[^ ^205^ ^]^ Teng et al. found that ginger exosomes can dramatically improve intestinal flora and protect the intestine from colitis.^[^ ^206^ ^]^ Extracellular vesicles derived from ginseng can regulate the polarization of macrophages through the TLR4‐MyD88 pathway and exert antitumor therapeutic effects.^[^ ^207^ ^]^ On the other hand, ginseng exosomes can also induce MSCs to differentiate into nerve cells through their active miRNAs, accelerating nerve regeneration and functional repair.^[^ ^208^ ^]^

In conclusion, extracellular vesicles usually have various immunomodulatory effects, with good biocompatibility and safety. The development of diagnosis and treatment methods based on extracellular vesicles has a high feasibility and clinical significance.

### 4.3 Cell‐based scaffold

Red blood cells (RBCs) are one of the most numerous cells in the human body and are mainly responsible for transporting oxygen to various tissues and organs. Previous studies have reported that RBCs have a regulatory effect on the innate immune system when the body develops diseases (e.g., inflammation and atherosclerosis), but the specific mechanism is still unclear.^[^ ^209^, ^210^ ^]^ The latest research found that RBCs can bind to the CpG‐DNA of some pathogens and mitochondria through the TLR9 receptor on the membrane surface, promoting the phagocytosis of RBCs and activating the innate immune system.^[^ ^211^, ^212^ ^]^ RBCs can also be used to deliver antigens and drugs for antitumor immunotherapy. It can bind antigens and deliver them to immune cells to enhance the immune response.^[^ ^213^, ^214^ ^]^

More interestingly, Fan et al. used RBCs to prepare a blood clot scaffold with controllable shape and size, which can be transplanted under the skin to establish a local antitumor niche (Figure 8C).^[^ ^215^ ^]^ The inside of the blood clot scaffold could recruit a large number of immune cells, including dendritic cells, macrophages, and T cells. The blood clot itself has the effect of activating immune cells, and a TLR9 agonist, GM‐CSF, and tumor‐associated antigen are also added to the blood clot, so this natural blood clot vaccine has achieved excellent therapeutic effects in a variety of tumor models. Furthermore, the gel prepared using RBCs combined with adjuvants can be used for the photothermal treatment of tumors and shows good therapeutic effects. In recent years, tumor vaccine therapy has become another research direction of tumor immunotherapy due to its great targeting and low cytotoxicity.^[^ ^216^, ^217^ ^]^ However, the tumor microenvironment is sophisticated, and its clinical efficacy is still very limited.^[^ ^218^, ^219^, ^220^ ^]^ RBCs obtained from the body have high biological safety, vast sources, relatively simple preparation, and high application potential. The design of a personalized treatment platform around RBCs for the treatment of more disease models is worthy of further exploration.

## 5 CONCLUSION AND OUTLOOK

In this review, we outline the latest developments in immunomodulatory biomaterials discovered in recent years, including metal oxides, silicon nanomaterials, carbon nanomaterials, synthetic polymer materials, biomacromolecule‐based materials, and cell‐derived materials. During the past few decades, biomaterials have often been designed as “inert carriers” for targeted delivery of drugs or immunomodulators to regulate systemic hyperactivation or hypoactivation. Although people have been optimizing biomaterials to reduce interactions with the immune system, the potential health risks of biomaterial retention is still a key issue that hinders its clinical transformation. In recent years, the choice of biomaterials has gradually changed, and biomaterials with immunomodulatory effects are becoming important tools in the field of biomedicine. To date, immunomodulatory biomaterials have provided many new opportunities in inflammation, infection, autoimmune diseases, tissue damage, and cancer treatment. With the deepening of research, the biological understanding of cell‐biomaterial interactions also provides the possibility for the development of new treatments. Although the current results show that immunomodulatory biomaterials have broad development prospects in future disease treatments, there are still some important issues that need to be considered and resolved before entering clinical research.

On the one hand, mechanisms of inflammation are still needed to explore. Inflammation is linked to the development of cancer and promotes all stages of tumorigenesis, while the prevailing view in cancer immunotherapy is that adjuvants improve outcomes of immunotherapy by promoting local inflammation. Further fundamental research is needed to explain this paradox more detailly. On the other hand, for the design of immunomodulatory biomaterials, one of the most significant problems for most inorganic materials is the degradability in the body, which may induce additional side effects. Furthermore, although synthetic or natural polymers have the advantages of wide applicability and bio‐degradability, most polymers have the problem of being highly complex, making them take a long time for the final clinical transformation. At last, although relevant clinical trials of cell‐derived bioactive materials (e.g., EVs) in the diagnosis and treatment of many diseases have been carried out, large‐scale industrial production still faces many challenges. There is still a lack of efficient processing, preservation, and extraction technologies. For example, the current extraction methods for exosomes have a relative low yields. Finally, the study of immunomodulatory biomaterials must examine whether endotoxin contamination was controlled. Some reported biomaterials may contain endotoxin which stimulates the immune cells activation, rather than biomaterials themselves. So contamination in biomaterials leads to the misinterpretation of immunomodulatory results which should draw attention in future investigations.

In summary, the future development of new immunomodulatory biomaterials is full of opportunities and challenges. Understanding the potential immunological effects of biomaterials can provide theoretical support for the safe use of biomaterials, and immunomodulatory biomaterials can also be used as immunotherapeutics for biomedical applications.

## CONFLICT OF INTEREST

The authors declare no conflict of interest.
